# Supplementary material for: A consistent budgeting of terrestrial carbon fluxes
Source: Nat Commun. 2024 Aug 28;15:7426. doi: 10.1038/s41467-024-51126-x (PMC11358497; doi:10.1038/s41467-024-51126-x)
Supplement: Supplementary file 1 — Supplementary Information [file 41467_2024_51126_MOESM1_ESM.pdf]

Supplementary materials: A consistent budgeting of terrestrial carbon fluxes

Lea Dorgeist[1], Clemens Schwingshackl*[1], Selma Bultan[1], Julia Pongratz[1,2]

[1] *Department of Geography, Ludwig-Maximilians-Universität, München, Germany*
[2] *Max Planck Institute for Meteorology, Hamburg, Germany*

***Correspondence: c.schwingshackl@lmu.de**

Supplementary Method 1 Mapping of DGVM PFTs to BLUE PFTs and BLUE cover types

BLUE distinguishes eleven PFTs within four land-cover types (primary land, secondary land, pasture, and cropland; see Hansis *et al.* [1]). As the number and definition of PFTs is not standardized in DGVMs, not all DGVM PFTs can directly be matched to the BLUE PFTs and the BLUE cover types. We thus take further steps to match the DGVM PFTs to the BLUE PFTs for the cover types primary land and secondary land and to the cover types pasture and cropland. The final mapping between DGVM PFTs to BLUE PFTs and BLUE cover types for all DGVMs can be found in Supplementary Data 1 and 2.

Supplementary Method 1.1 Mapping of primary and secondary land

1. **Forest**: BLUE considers six forest PFTs, distinguishing between their geographical location (tropical or temperate/boreal), their types of leaves (needleleaf or broadleaf), and their phenology (evergreen or deciduous). In some DGVMs, several forest PFTs can be directly matched to the BLUE PFTs (Supplementary Data 1). In other cases, the geographical extent of a PFT is larger in the DGVM than in BLUE, and we thus apply a spatial mask to fit its spatial extent to the spatial extent of the BLUE PFT (Supplementary Fig. 13). For DGVMs that have a more detailed distinction of forest PFTs than BLUE, the respective PFTs are aggregated into one PFT by taking the annual mean of the carbon densities of the involved PFTs to match the respective BLUE PFT. After matching the DGVM PFTs to the BLUE PFTs, we calculate the annual global carbon density for each PFT by dividing the global sum of carbon stocks in each PFT (cVegpft $*$ gridarea $*$ landCoverFrac$_{pft}$) by the global area of each PFT (gridarea $*$ landCoverFrac$_{pft}$) using the data from the TRENDY S2 simulation. Here, cVegpft is the carbon content of the PFT in each grid cell, gridarea is the area of each grid cell, and landCoverFrac$_{pft}$ is the fraction of each grid cell covered by the respective PFT.

2. **Shrub**: Further, BLUE defines two shrub PFTs (raingreen and summergreen shrubs). While some DGVMs lack the distinction of raingreen and summergreen shrubs (YIBs and CABLE-POP), other DGVMs do not further distinguish shrubs at all. For the former case, we apply a spatial mask to the respective shrub PFTs in YIBs and CABLE-POP to match them to the spatial extent of raingreen and summergreen shrubs in BLUE (see Supplementary Fig. 13). Afterwards we calculate the annual global carbon densities for both shrub PFTs, following the same approach as for forest PFTs. For the latter case (DGVMs without shrub PFTs), we follow the approach of Poulter *et al.* [2] (see their Table 2), who provide a cross-walking table with the coverage of each PFT within different types of land-cover classes based on the United Nations Land Cover Classification System (UNLCCS). We use the cross-walking table to construct the carbon densities for raingreen and summergreen shrubs by weighting the annual global carbon densities (which we calculate by the same approach as used for forests) of the involved PFTs (i.e., forest and grass PFTs) according to their distribution within the UNLCCS land-cover class "Shrub deciduous" (Supplementary Data 2).

3. **Tundra**: For DGVMS that do not have a dedicated PFT for Tundra, we use the C3 grass PFT and fit it to the spatial extent of the Tundra PFT in BLUE by applying a spatial mask (Supplementary

Fig. 13). The annual global carbon density for Tundra is then calculated by the same approach as used for forests.

Supplementary Method 1.2 Mapping of pasture and cropland

For the BLUE cover types pasture and cropland, the respective pasture and cropland PFTs of DGVMs are used. If a DGVM does not consider pasture nor cropland, the grass PFTs are used to represent cropland and pasture. If a DGVM has cropland but not pasture, the grass PFTs are used to represent pasture. For DGVMs that have a more detailed distinction of cropland and/or pasture types, the respective PFTs are aggregated by taking the annual mean of the carbon densities of the involved PFTs. An overview of which DGVM PFTs are used for pasture and cropland can be found in Supplementary Data 2.

The annual global carbon densities are then calculated following the same approach as for forest PFTs. All DGVMs distinguish between the C3 and C4 photosynthetic pathway for pasture and cropland, which is not the case for BLUE, where pasture and cropland are considered as land-cover types with PFT-dependent carbon densities ([1]). To account for the distribution of C3 and C4 plants within each BLUE PFT, we calculate the total land-cover change of C3 and C4 pasture/cropland within each BLUE PFT from the TRENDY S3 simulation (which includes land-use change).

First, the land-cover fractions of the involved DGVM PFTs are regridded to the resolution of BLUE using conservative remapping. Second, the total land-cover change of C3 and C4 plants is estimated as the average change of their land-cover fraction between 1700-1709 and 2012-2021. Third, only grid cells with positive changes are included in further analysis, as this represents grid cells with historical increases in C3 or C4 pasture/cropland extent. The total (positive) land-cover change of C3 and C4 plants is multiplied with the land-cover fraction of each BLUE PFT to only include changes within the considered BLUE PFT. Finally, the share of C3 and C4 plants on the total expansion of cropland/pasture is calculated. This resembles whether and to what extent the natural vegetation is replaced by C3 and C4 plant types (e.g., a share of 32% of C3 pasture in PFT1 means that 32% of the land cover of PFT1 "Tropical Evergreen Forest" becomes C3 pasture). The annual global carbon densities for pasture and cropland are weighted according to the share of C3 and C4 plants on the cropland/pasture expansion.

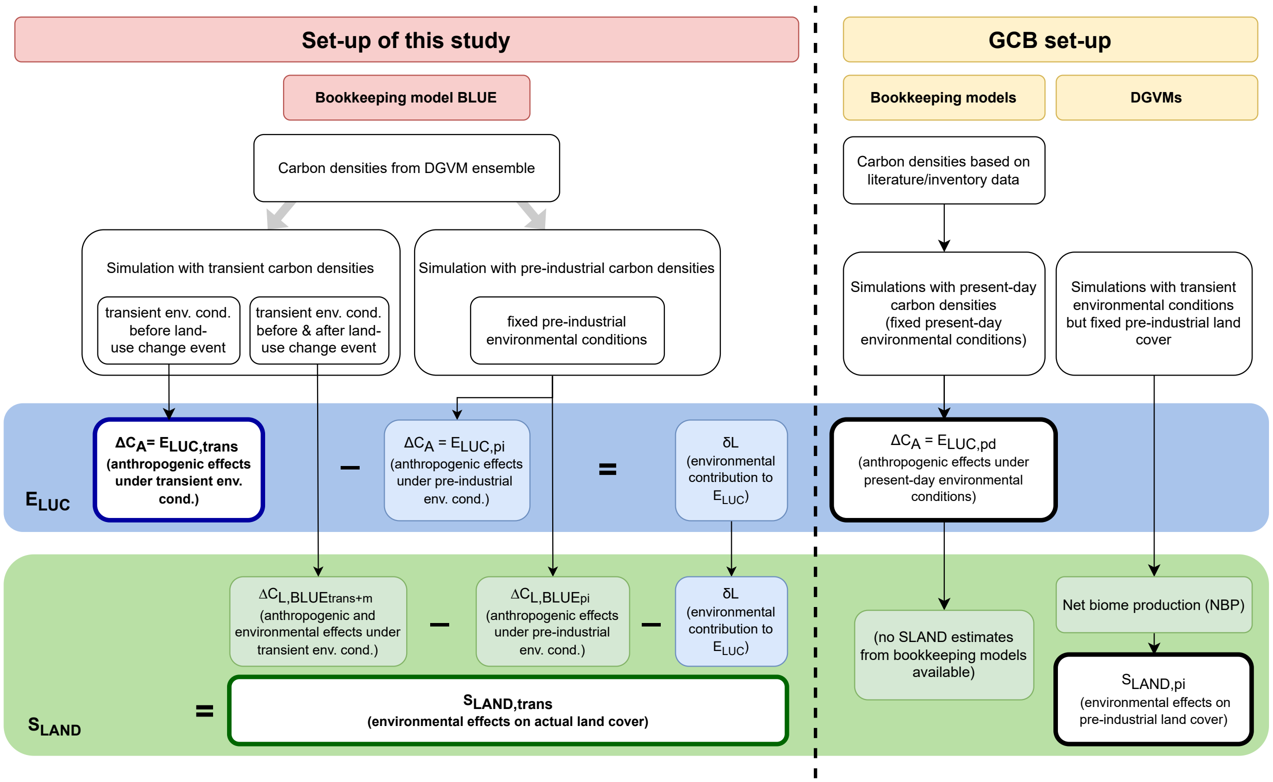

Supplementary Fig. 1: **Schematic overview of the set-ups used in this study and in the Global Carbon Budget (GCB) to estimate E_{LUC} (highlighted in blue) and S_{LAND} (highlighted in green).** In our study, E_{LUC} and S_{LAND} are estimated by the bookkeeping model BLUE, whereas the GCB uses three bookkeeping models to estimate E_{LUC} and DGVMs to estimate S_{LAND}. In our study, we integrate transient DGVM carbon densities into BLUE. The BLUE simulation using transient carbon densities yields $E_{LUC,trans}$, which includes the effect of transient environmental changes on E_{LUC}, and the anthropogenic and environmental effects under transient environmental conditions ($\Delta C_{L,BLUE_{trans+m}}$). The BLUE simulation using pre-industrial carbon densities yields $E_{LUC,pi}$, which is used to estimate the environmental contribution to E_{LUC} (δ_L, difference between $E_{LUC,trans}$ and $E_{LUC,pi}$), and the anthropogenic effects under pre-industrial environmental conditions ($\Delta C_{L,BLUE_{pi}}$). Subtracting $\Delta C_{L,BLUE_{pi}}$ and δ_L from $\Delta C_{L,BLUE_{trans+m}}$ yields $S_{LAND,trans}$, i.e. S_{LAND} under actual transiently changing land cover. The desired E_{LUC} estimate in our set-up is indicated by a box with thick, blue frame and the desired S_{LAND} estimate by a box with thick, green frame. The E_{LUC} and S_{LAND} estimates calculated with the GCB set-up are highlighted with thick, black frames. These have the shortcomings of being calculated under present-day environmental conditions (E_{LUC}) or under pre-industrial land cover (S_{LAND}). ΔC_A is the annual change of the atmospheric carbon pool and ΔC_L is the combined annual change of the biomass, soil and product carbon pools.

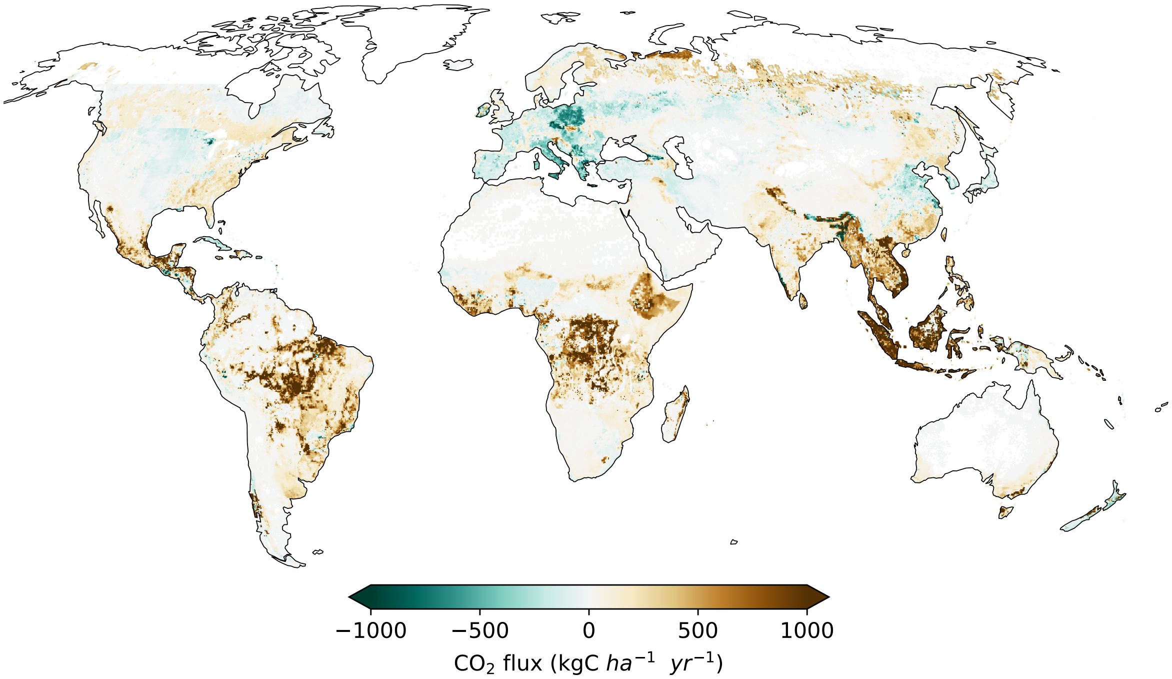

Supplementary Fig. 2: **Emissions from land-use change under transient environmental conditions ($E_{LUC,trans}$).** Global map of annual land-use change emissions averaged over 2012-2021 calculated with the bookkeeping model BLUE applying transient carbon densities. Positive values indicate carbon emissions and negative values indicate carbon removals.

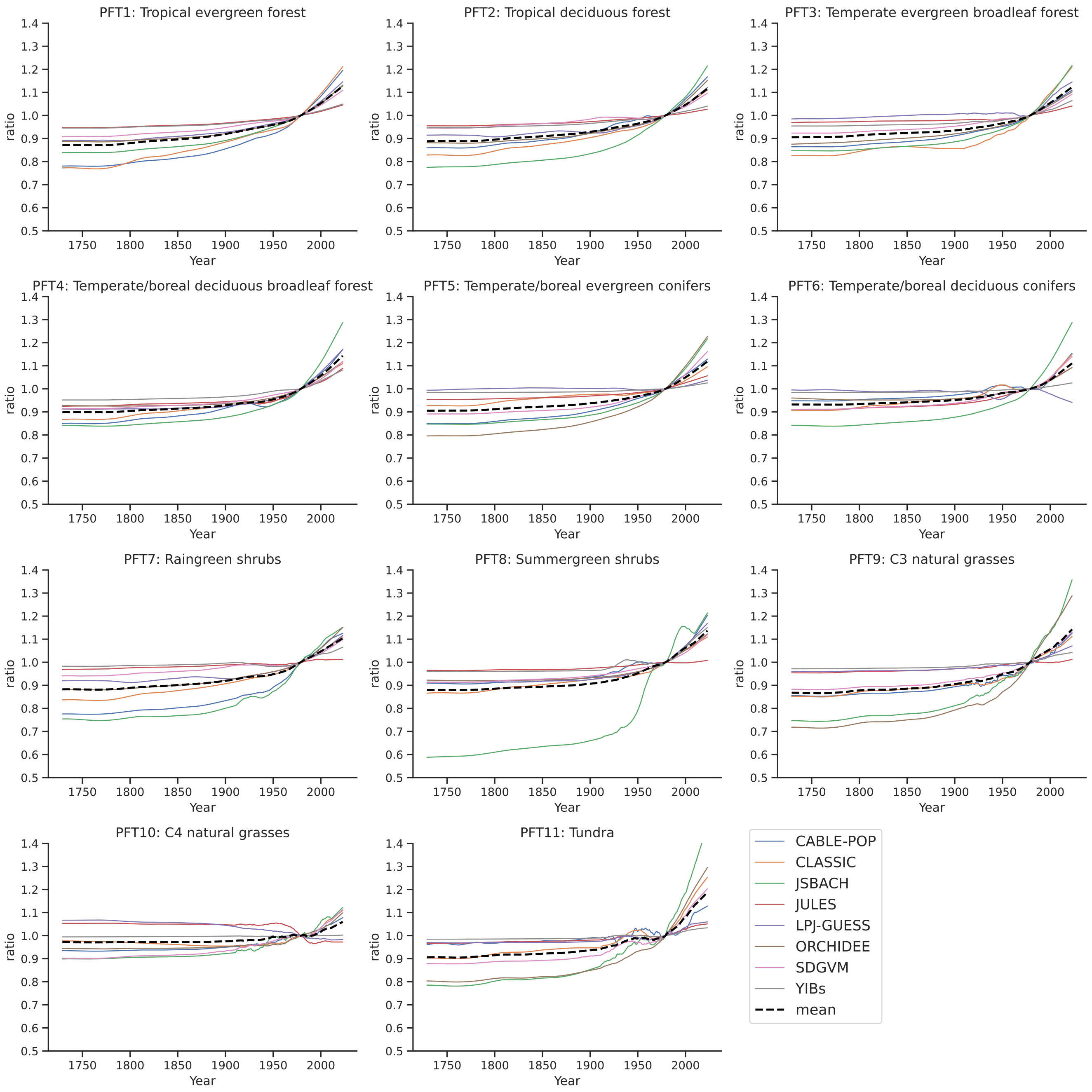

Supplementary Fig. 3: **Time series of carbon density ratios for vegetation relative to the year 1980.** Subplots show global averages of carbon density ratios for 11 plant functional types (PFTs) used in BLUE for primary/secondary land (see Supplementary Data 1). Different lines show the ratios for different DGVMs and their mean is shown as black dashed line. Ratios are based on the year 1980 as this is the year where the default BLUE carbon densities approximately originate from (see Methods). DGVM PFTs are mapped to BLUE PFTs and BLUE cover types (see Supplementary Method 1, Supplementary Data 1 and 2). Carbon density ratios for pasture and crop (whose ratios vary over the 11 PFTs) are not shown.

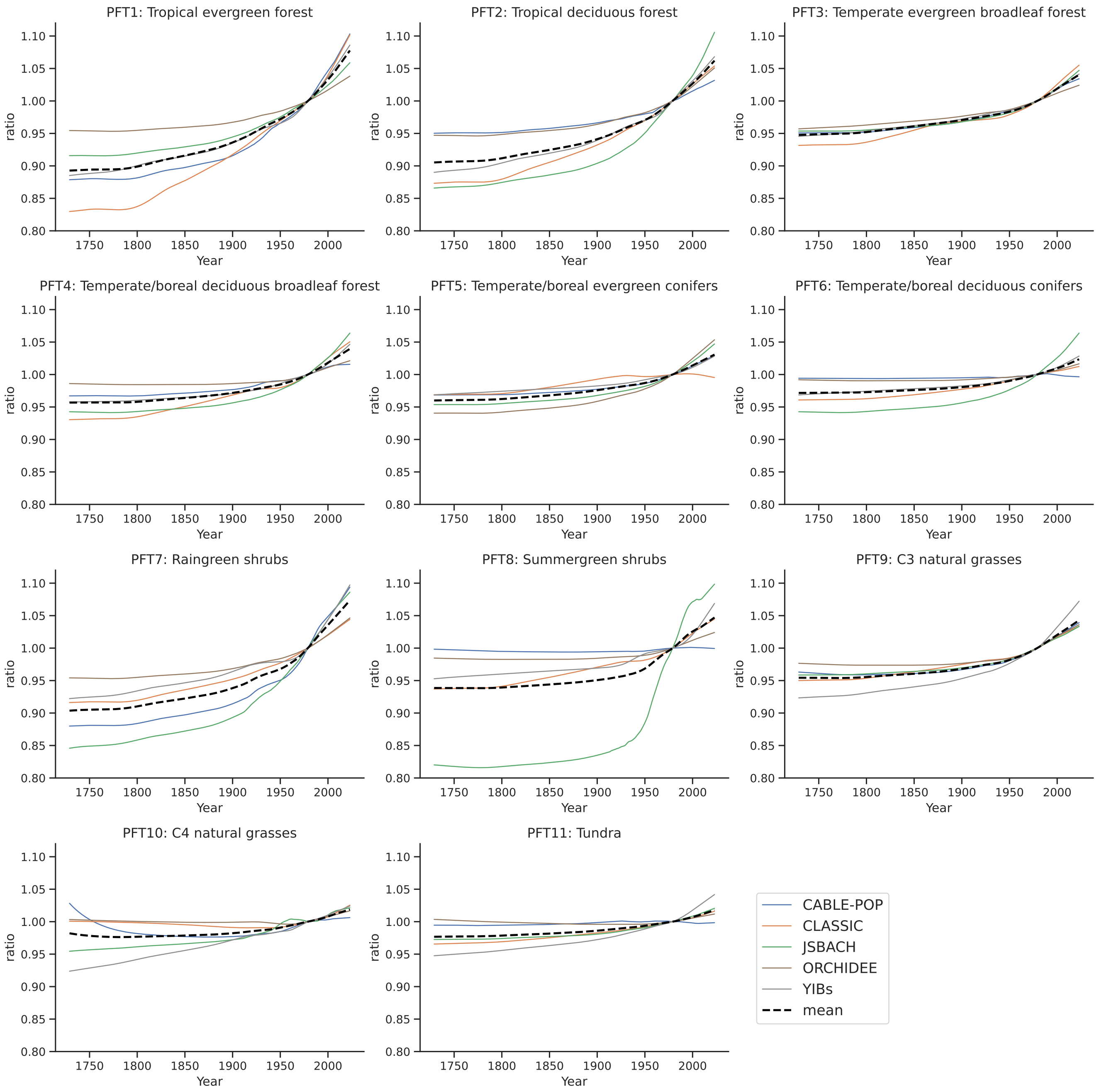

Supplementary Fig. 4: **As in Supplementary Fig. 3 but for soil carbon instead of vegetation carbon.** Subplots show global averages of carbon density ratios for 11 plant functional types (PFTs) used in BLUE for primary/secondary land (see Supplementary Data 1). Different lines show the ratios for different DGVMs and their mean is shown as black dashed line. Ratios are based on the year 1980 as this is the year where the default BLUE carbon densities approximately originate from (see Methods). DGVM PFTs are mapped to BLUE PFTs and BLUE cover types (see Supplementary Method 1, Supplementary Data 1 and 2). Carbon density ratios for pasture and crop (whose ratios vary over the 11 PFTs) are not shown.

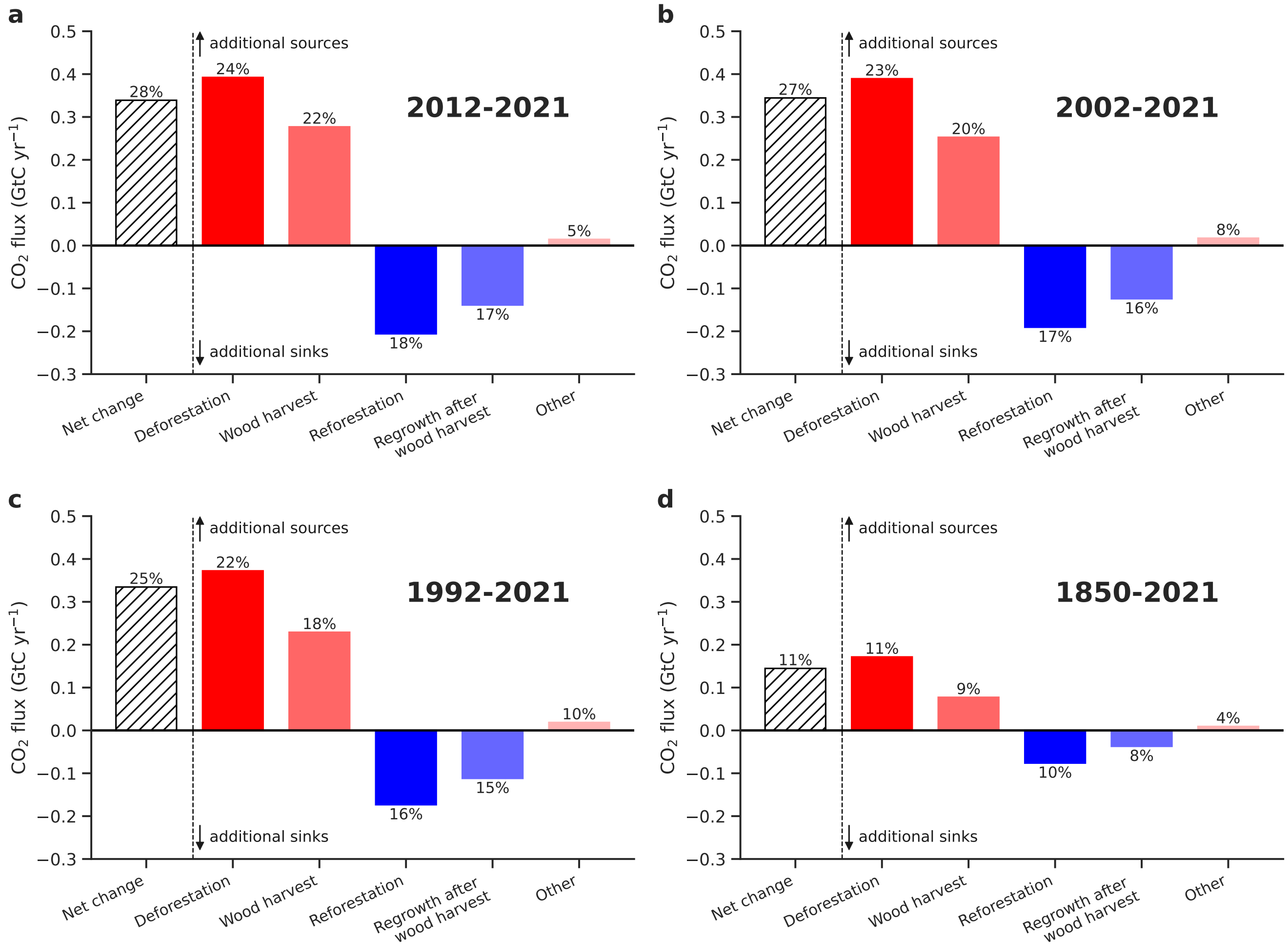

Supplementary Fig. 5: **Environmental contribution to E_{LUC} (additional sources or sinks) of major land-use transitions and land-management types for different time periods: (a) 2012-2021, (b) 2002-2021, (c) 1992-2021, (d) 1850-2021.** The environmental contribution to E_{LUC} is the difference between E_{LUC} under transient environmental conditions ($E_{LUC,trans}$) and under pre-industrial environmental conditions ($E_{LUC,pi}$). Values are averaged over four different time periods as absolute values and as percentage change of each component relative to pre-industrial conditions.

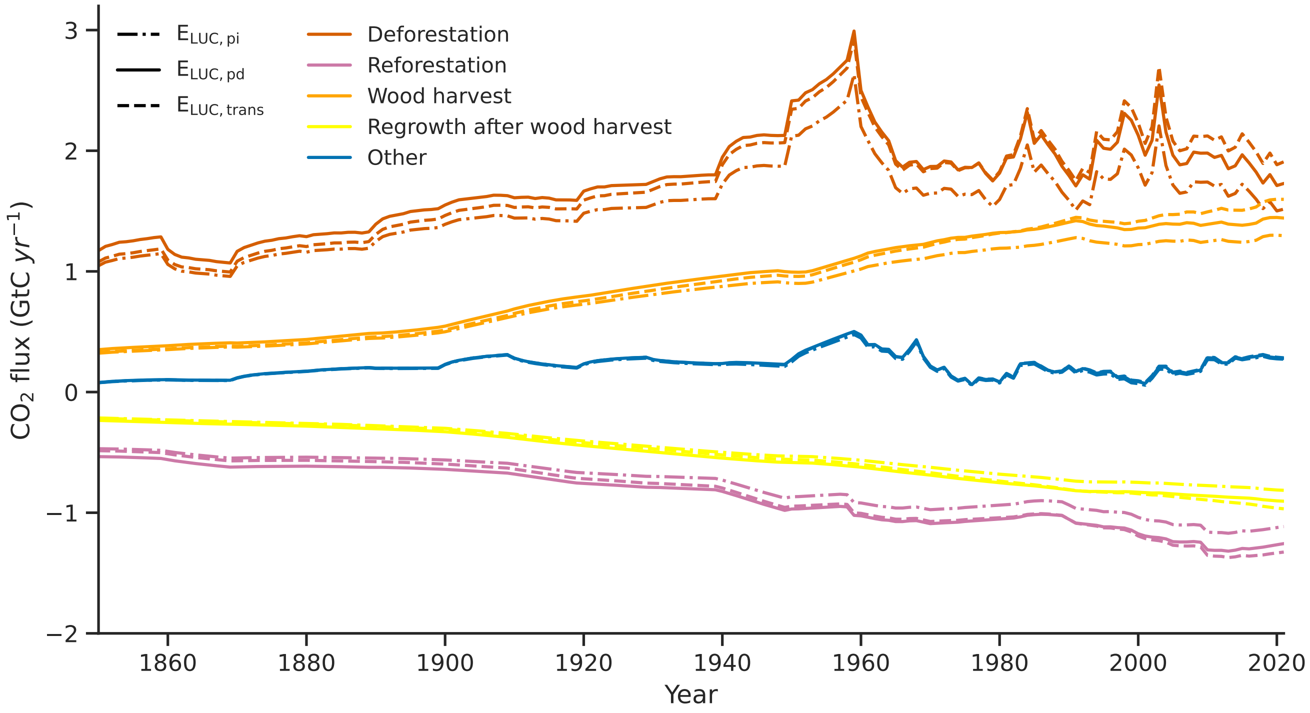

Supplementary Fig. 6: **Effects of different environmental conditions on land-use change emission estimates of major land-use transition types.** Global annual land-use change emissions (E_{LUC}) from three simulations applying pre-industrial ($E_{LUC,pi}$), present-day ($E_{LUC,pd}$), and transient ($E_{LUC,trans}$) carbon densities split into major land-use transition types (deforestation, reforestation, wood harvest, regrowth after wood harvest, other transitions).

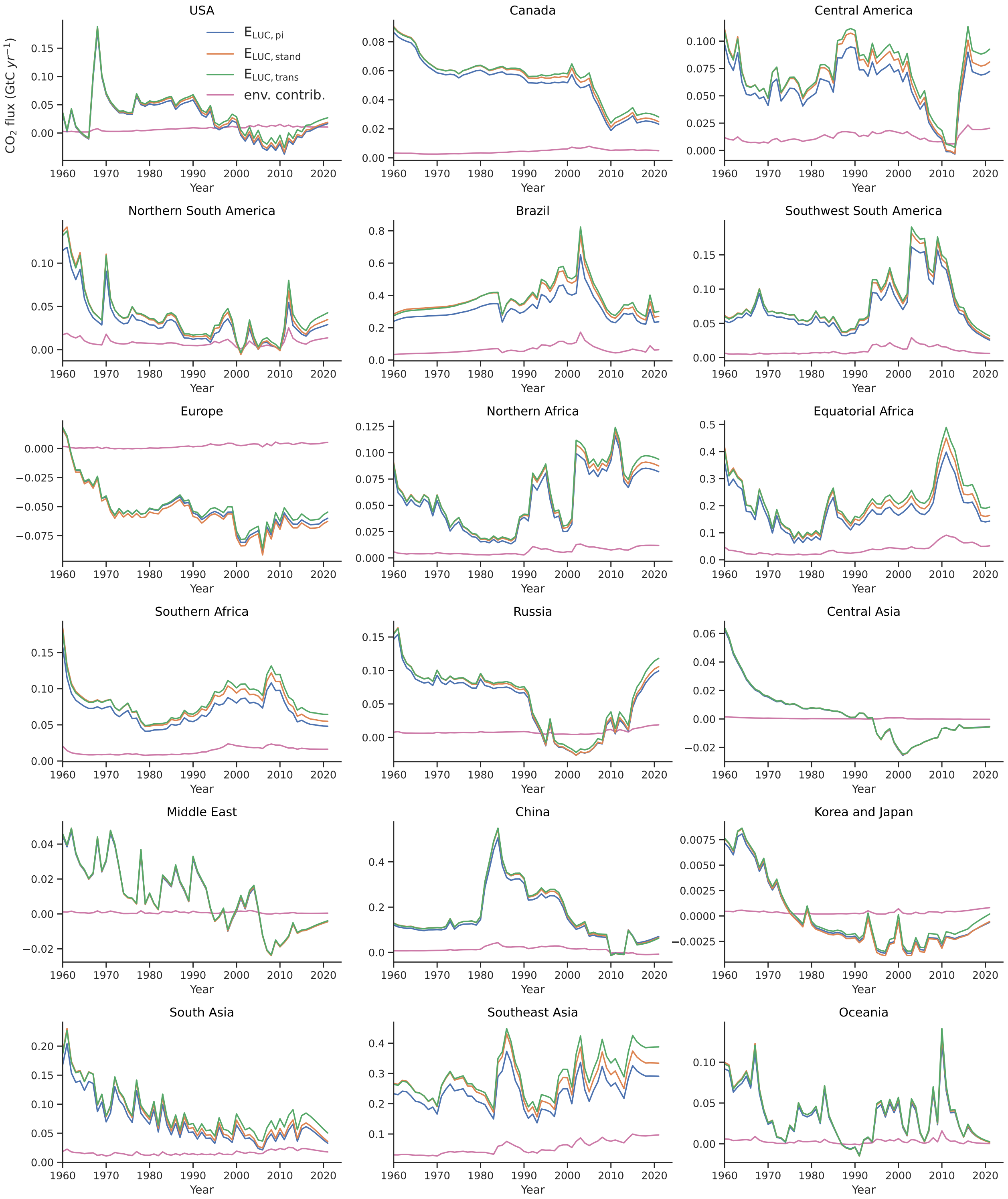

Supplementary Fig. 7: **Regional effects of different environmental conditions on land-use change emission estimates.** Regional estimates of annual land-use change emissions from three BLUE simulations applying pre-industrial ($E_{LUC,pi}$), present-day ($E_{LUC,pd}$) and transient ($E_{LUC,trans}$) carbon densities. Our definition of regions is based on the Regional Carbon Cycle Assessment and Processes 2 (RECCAP2) project ([3]). Note that the scale of the y-axis differs across the shown regions.

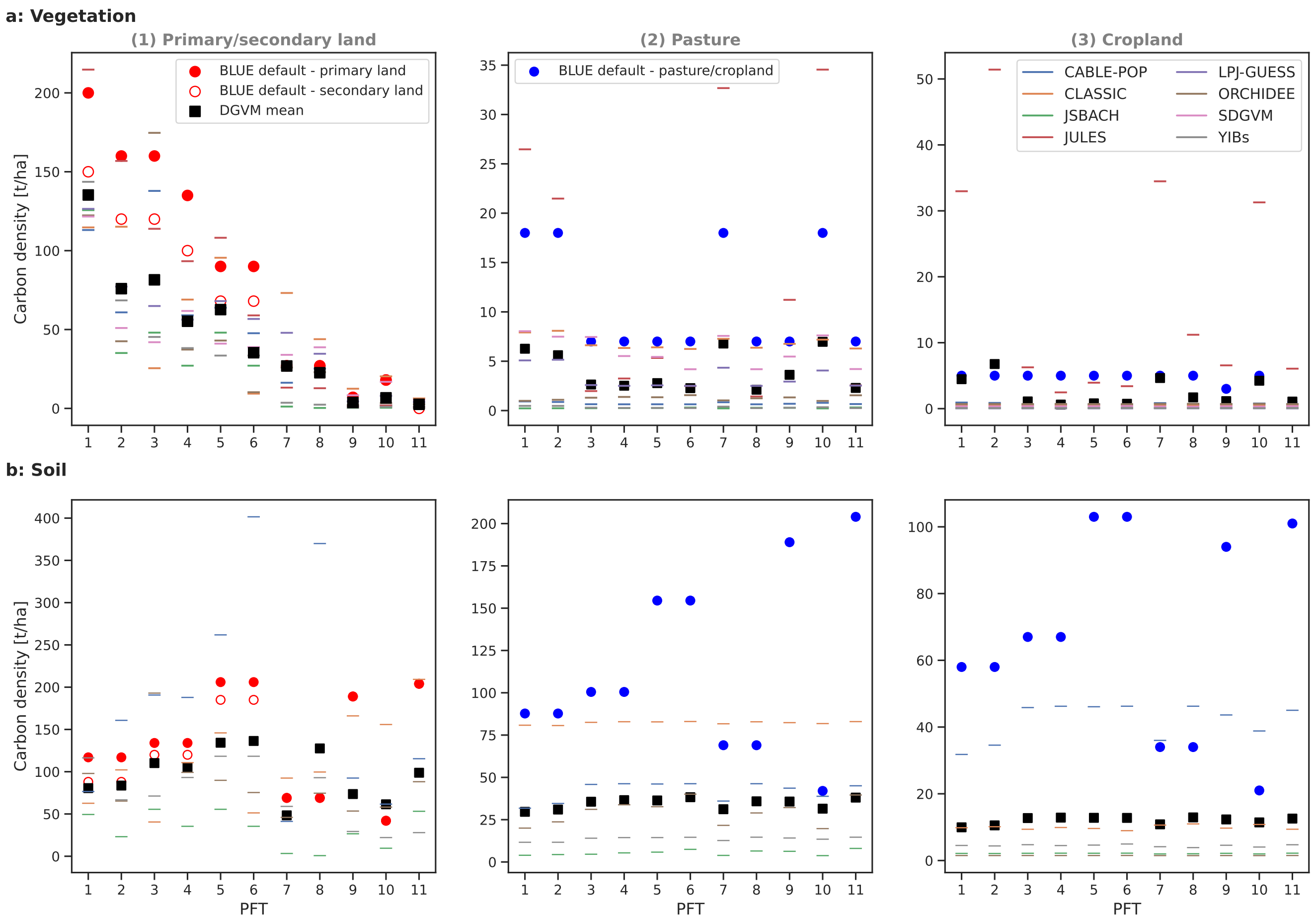

Supplementary Fig. 8: **Comparison of BLUE carbon densities and DGVM carbon densities.** Subplots show carbon densities of BLUE and DGVMs for vegetation (a) and soil (b) for the cover types primary/secondary land (1), pasture (2), and cropland (3) for the eleven plant functional types (PFT) of BLUE (see Supplementary Data 1). BLUE carbon densities are taken from Hansis *et al.* [1] based on Houghton *et al.* [4]. DGVM values are global averages over 1970-1990 to be comparable to the BLUE carbon densities from ~1980. DGVM PFTs are mapped to BLUE PFTs and to BLUE cover types (see Supplementary Method 1, Supplementary Data 1 and 2).

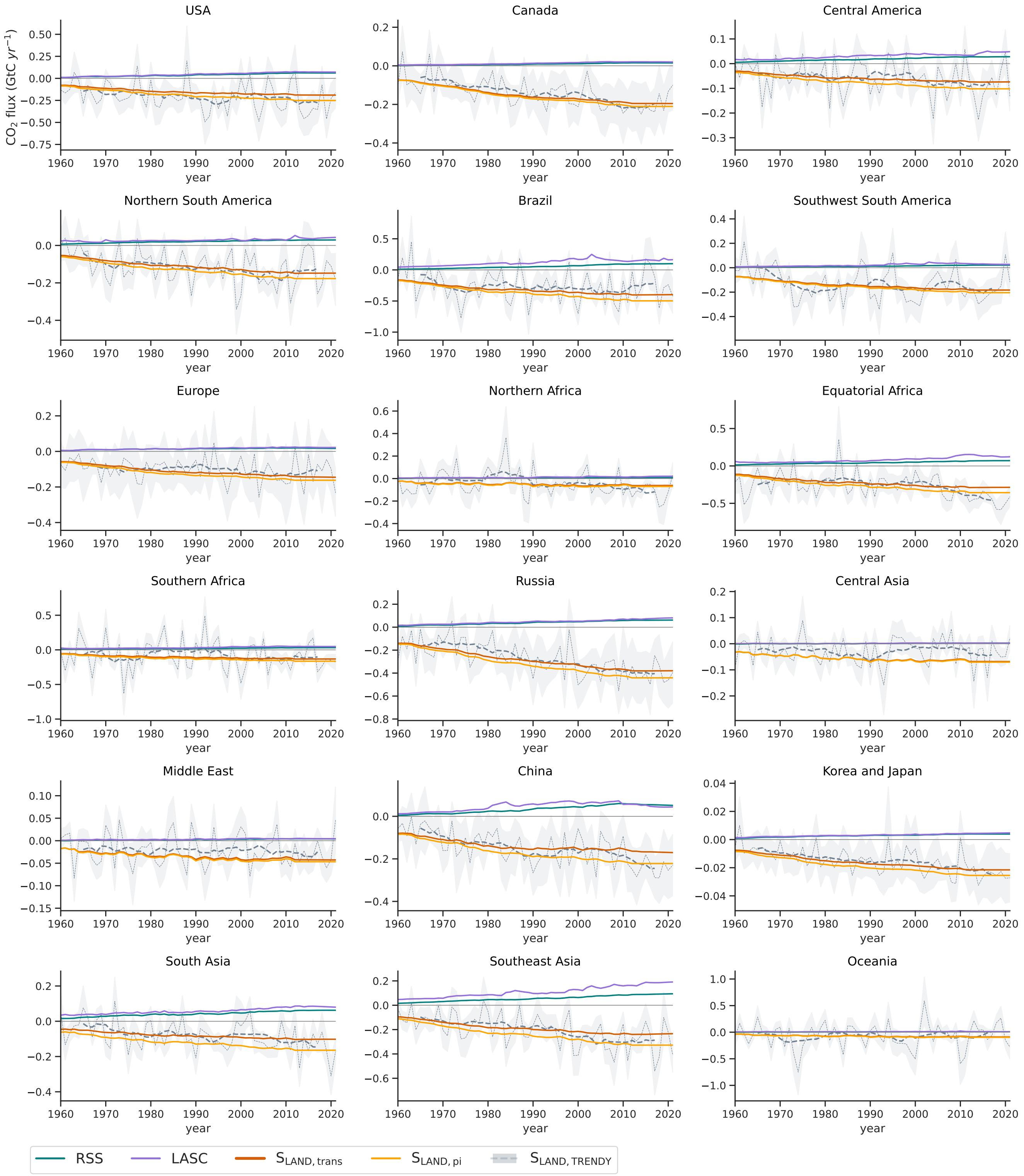

Supplementary Fig. 9: **As in Fig. 2 in the manuscript but for regional estimates.** Our definition of regions is based on the Regional Carbon Cycle Assessment and Processes 2 (RECCAP2) project ([3]). Note that the scale of the y-axis differs across the shown regions.

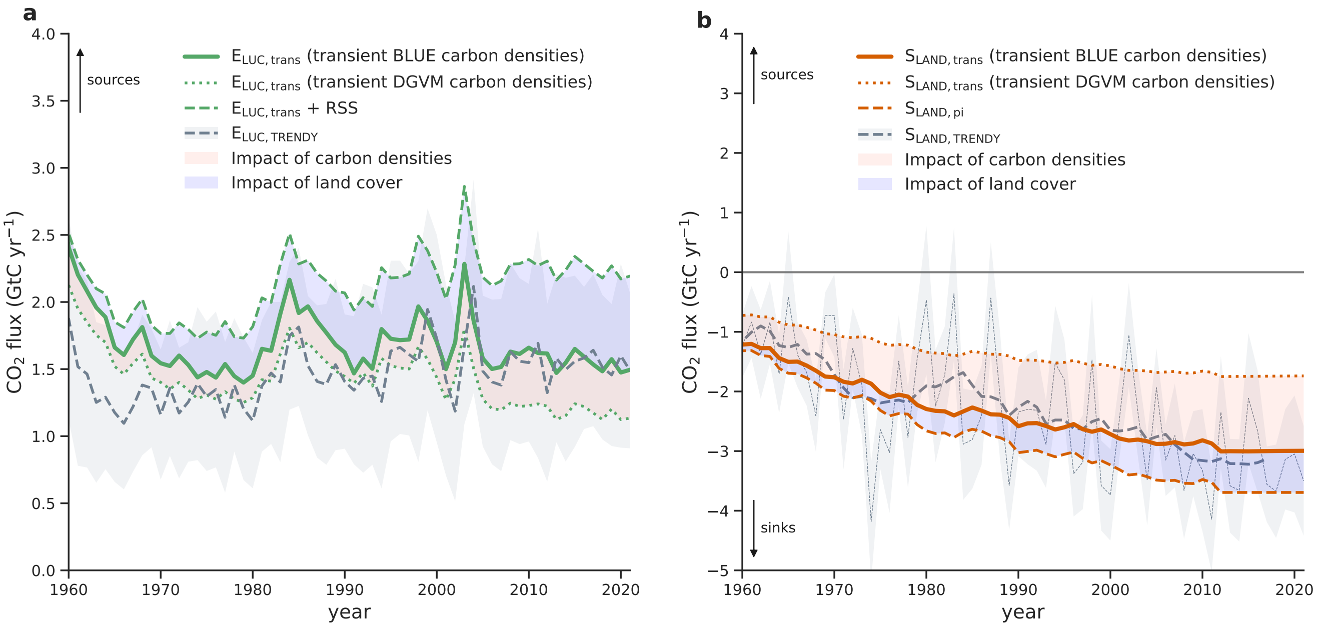

Supplementary Fig. 10: **Impact of carbon densities and land cover on E_{LUC} and S_{LAND} estimates.** (a) Global annual land-use change emissions (E_{LUC}, averaged over 2012-2021) from the bookkeeping model BLUE using transient BLUE carbon densities ($E_{LUC,trans}$, solid green line, as in Fig. 1) and using transient DGVM carbon densities (dotted green line). The difference between these simulations illustrates the impact of different absolute carbon densities (see Supplementary Fig. 8 for carbon density values). Yet more important are conceptual differences: $E_{LUC,trans}$ + RSS (dashed green line) conceptually equals $E_{LUC,TRENDY}$ (i.e., the E_{LUC} estimate based on Dynamic Global Vegetation Models (DGVMs) from the TRENDY project; dashed grey line) as the environmental contribution to E_{LUC} included in $E_{LUC,trans}$ plus RSS equals the LASC contained in $E_{LUC,TRENDY}$ (RSS = replaced sources and sinks; LASC = loss of additional sink capacity). (b) BLUE estimates of the global annual natural land sink estimated on actual, transient land cover using transient BLUE carbon densities ($S_{LAND,trans}$, solid orange line) and using transient DGVM carbon densities ($S_{LAND,trans}$, dotted orange line). $S_{LAND,pi}$ conceptually equals $S_{LAND,TRENDY}$ (dashed grey line) as both estimates are based on pre-industrial land cover. The red and blue shaded areas indicate the impact of different land-cover assumptions and the impact of different carbon densities on the E_{LUC} and S_{LAND} estimates. Globally, the impacts of carbon densities and land cover largely offset each other, explaining the similar estimates of E_{LUC} ($E_{LUC,trans}$ and $E_{LUC,TRENDY}$) and S_{LAND} ($S_{LAND,trans}$ and $S_{LAND,TRENDY}$).

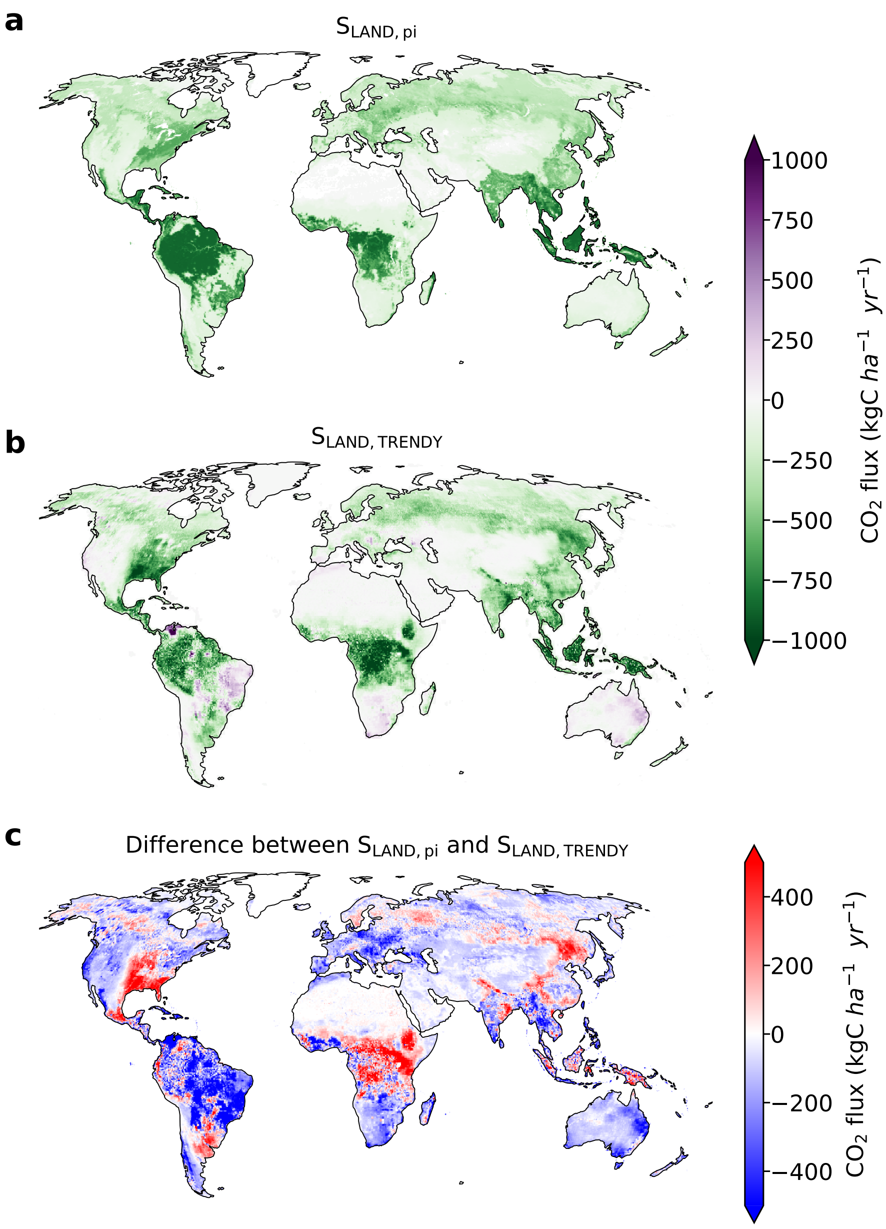

Supplementary Fig. 11: **Global maps of the natural land sink from BLUE and TRENDY under pre-industrial land cover.** Spatial distribution of the natural land sink (S_{LAND}) averaged over 2012-2021 derived from (a) BLUE ($S_{LAND,pi}$) and (b) TRENDY ($S_{LAND,TRENDY}$) under pre-industrial land cover. (c) Spatial distribution of the difference between (a) and (b). Negative values indicate that $S_{LAND,pi}$ is larger than $S_{LAND,TRENDY}$ and vice versa. The difference results from model-intrinsic differences between BLUE and the TRENDY models (e.g., related to different carbon densities, see Supplementary Fig. 8 and Supplementary Fig. 10).

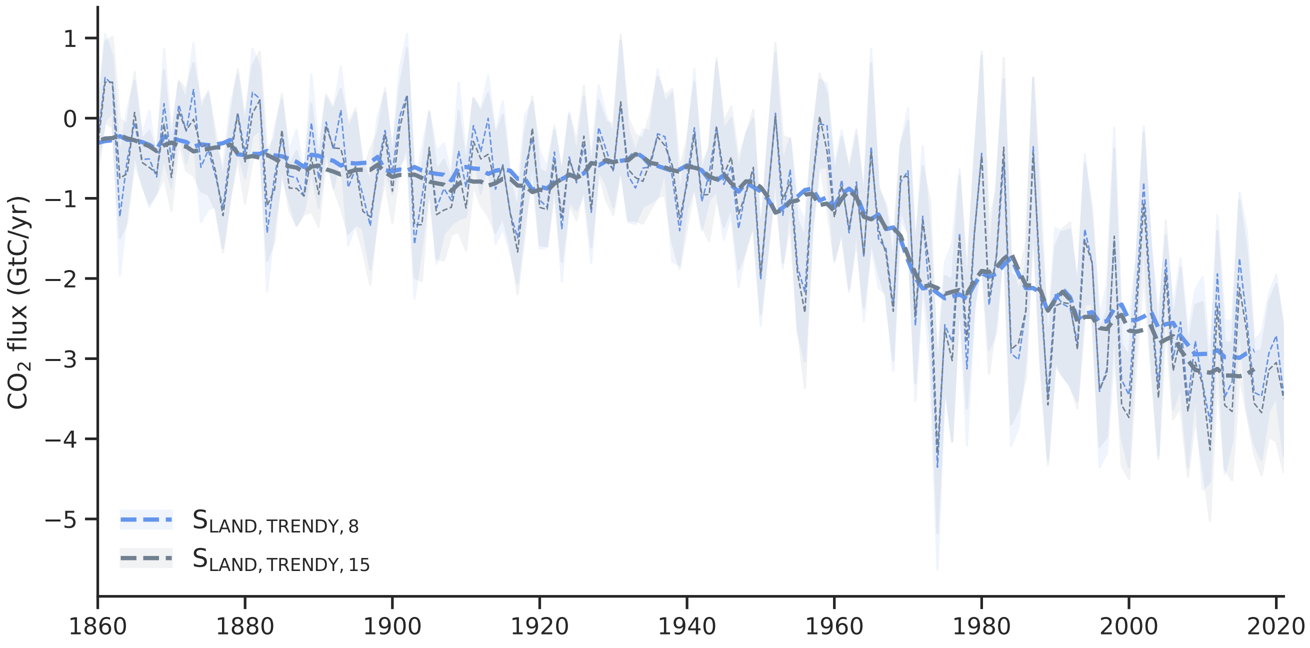

Supplementary Fig. 12: **Impact of the number of DGVMs on the estimate of the natural land sink.** Global estimate of the natural land sink (S_{LAND}) using 15 DGVMs ($S_{LAND,TRENDY,15}$) or 8 DGVMs ($S_{LAND,TRENDY,8}$) from TRENDY. The latter selection (8 DGVMs) includes the DGVMs that are used to scale the default BLUE carbon densities (see Methods). Thin lines show annual values, thick lines show 10-year moving averages. Uncertainties (shaded areas) are shown as one standard deviation across DGVM estimates.

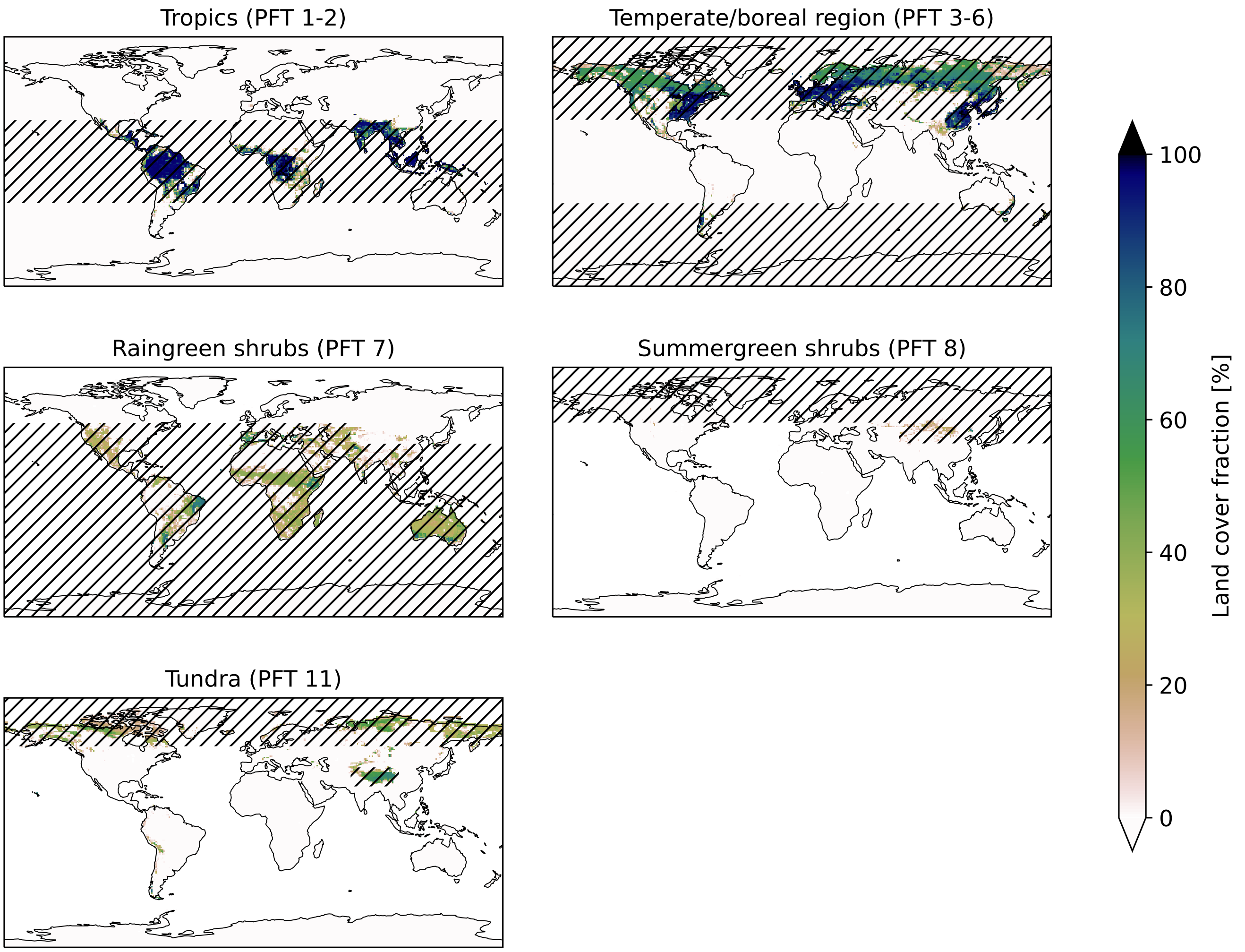

Supplementary Fig. 13: **Spatial masks used to match the spatial extent of some DGVM PFTs to the respective BLUE PFTs.** Hatching denotes the spatial extent of the mask. The colors in the background indicate the land-cover fraction of the indicated PFTs in BLUE. The application of these masks is described in Supplementary Method 1.

Supplementary Table 1: **Environmental and land-use components of the terrestrial carbon budget for different time periods.** All values are estimated by BLUE, except for the cumulative net land flux estimate from $\delta^{13}C$ records which is derived from Joos *et al.* [5]. Mean values are in GtC yr^{-1}, cumulative values are in GtC. PD stands for present-day, and PI for pre-industrial. See Table 1 and Table 2 in the main manuscript for definition of variables.

		E_{LUC} (transient)	E_{LUC} (PI)	E_{LUC} (PD)	S_{LAND} (transient)	S_{LAND} (PI)	Net land flux	LASC	RSS	Env. contrib.
Mean	**2012-2021**	1.5	1.2	1.4	-3.0	-3.7	-1.2	1.0	0.7	0.3
(GtC yr^{-1})		[1.5, 1.7]	[1.1, 1.3]		[-3.9, -2.2]	[-5.2, -2.6]	[-2.1, -0.5]	[0.5, 1.7]	[0.3, 1.3]	[0.2, 0.6]
	**2009-2018**	1.6	1.2	1.4	-3.0	-3.6	-1.2	1.0	0.7	0.3
		[1.5, 1.7]	[1.1, 1.3]		[-3.8, -2.2]	[-5.1, -2.6]	[-2.0, -0.4]	[0.5, 1.7]	[0.3, 1.3]	[0.2, 0.6]
	**2002-2021**	1.6	1.3	1.4	-2.9	-3.6	-1.1	1.0	0.7	0.3
		[1.5, 1.7]	[1.2, 1.3]		[-3.8, -2.1]	[-5.0, -2.5]	[-1.9, -0.3]	[0.5, 1.7]	[0.3, 1.2]	[0.2, 0.6]
	**1992-2021**	1.6	1.3	1.5	-2.8	-3.4	-1.0	0.9	0.6	0.3
		[1.6, 1.7]	[1.2, 1.4]		[-3.6, -2.0]	[-4.7, -2.4]	[-1.7, -0.2]	[0.4, 1.6]	[0.2, 1.1]	[0.2, 0.5]
Cumulative	**1850-1995**	210	194	214	-150	-167	78	33	17	16
(GtC)		[205, 214]	[181, 204]		[-200, -106]	[-232, -118]	[24, 123]	[13, 55]	[2, 32]	[9, 27]
	$\delta^{13}C$	-	-	-	-	-	31	-	-	-
	**1850-1995**						[-26, 88]			

Supplementary Reference

1. Hansis, E., Davis, S. J. & Pongratz, J. Relevance of methodological choices for accounting of land use change carbon fluxes. *Global Biogeochemical Cycles* **29,** 1230–1246. ISSN: 0886-6236, 1944-9224. https://onlinelibrary.wiley.com/doi/10.1002/2014GB004997 (2023) (Aug. 2015).

2. Poulter, B. *et al.* Plant functional type classification for earth system models: results from the European Space Agency's Land Cover Climate Change Initiative. *Geoscientific Model Development* **8,** 2315–2328. ISSN: 1991-9603. https://gmd.copernicus.org/articles/8/2315/2015/ (2023) (July 31, 2015).

3. Tian, H. *et al.* Global soil nitrous oxide emissions since the preindustrial era estimated by an ensemble of terrestrial biosphere models: Magnitude, attribution, and uncertainty. *Global Change Biology* **25,** 640–659. ISSN: 1365-2486. https://onlinelibrary.wiley.com/doi/abs/10.1111/gcb.14514 (2024) (2019).

4. Houghton, R. A. *et al.* Changes in the Carbon Content of Terrestrial Biota and Soils between 1860 and 1980: A Net Release of CO2 to the Atmosphere. *Ecological Monographs* **53,** 235–262. ISSN: 0012-9615, 1557-7015. https://onlinelibrary.wiley.com/doi/10.2307/1942531 (2023) (Sept. 1983).

5. Joos, F., Meyer, R., Bruno, M. & Leuenberger, M. The variability in the carbon sinks as reconstructed for the last 1000 years. *Geophysical Research Letters* **26,** 1437–1440. ISSN: 1944-8007. https://onlinelibrary.wiley.com/doi/abs/10.1029/1999GL900250 (2024) (1999).
