# Supplementary material for: A consistent budgeting of terrestrial carbon fluxes
Source: Nat Commun. 2024 Aug 28;15:7426. doi: 10.1038/s41467-024-51126-x (PMC11358497; doi:10.1038/s41467-024-51126-x)
Supplement: Supplementary file 3 — Description of Additional Supplementary Files [file 41467_2024_51126_MOESM3_ESM.pdf]

Description of Additional Supplementary Files

File name: Supplementary Data 1
Description: **Numbers and names of BLUE PFTs and DGVM PFTs relevant to scale the BLUE carbon densities.** DGVM PFTs are subsequently mapped to BLUE PFTs and cover types, see Supplementary Table 1.

File name: Supplementary Data 2
Description: **Mapping of DGVM PFTs to BLUE PFTs and to BLUE cover types Pasture and Cropland**. The numbers indicate the PFTs as they are listed in Supplementary Data 1. If multiple DGVM PFTs are mapped to one BLUE PFT, the respective DGVM PFTs are aggregated by taking the annual mean of the global carbon densities. The full description of how DGVM PFTs are mapped to BLUE PFTs is available in Supplementary Method 1.

* Carbon densities are calculated by weighting the timeseries of respective forest and grass PFTs based on Poulter et al. [2], see their Table 2. For some DGVMs, which do not consider bare soil, the sum of percentages might not add up to 100%.
** C3/C4 is weighted depending on the change in the land-cover fraction within each PFT (see Supplementary Method 1.2).
